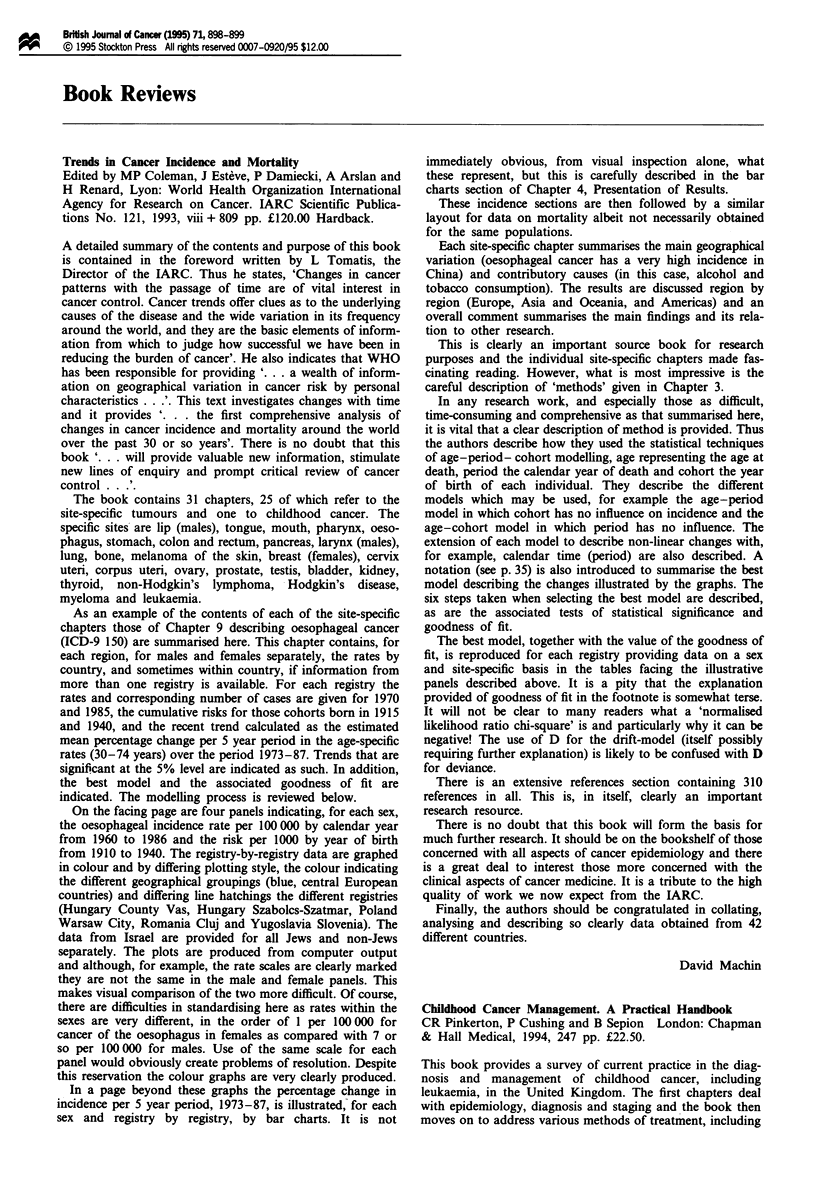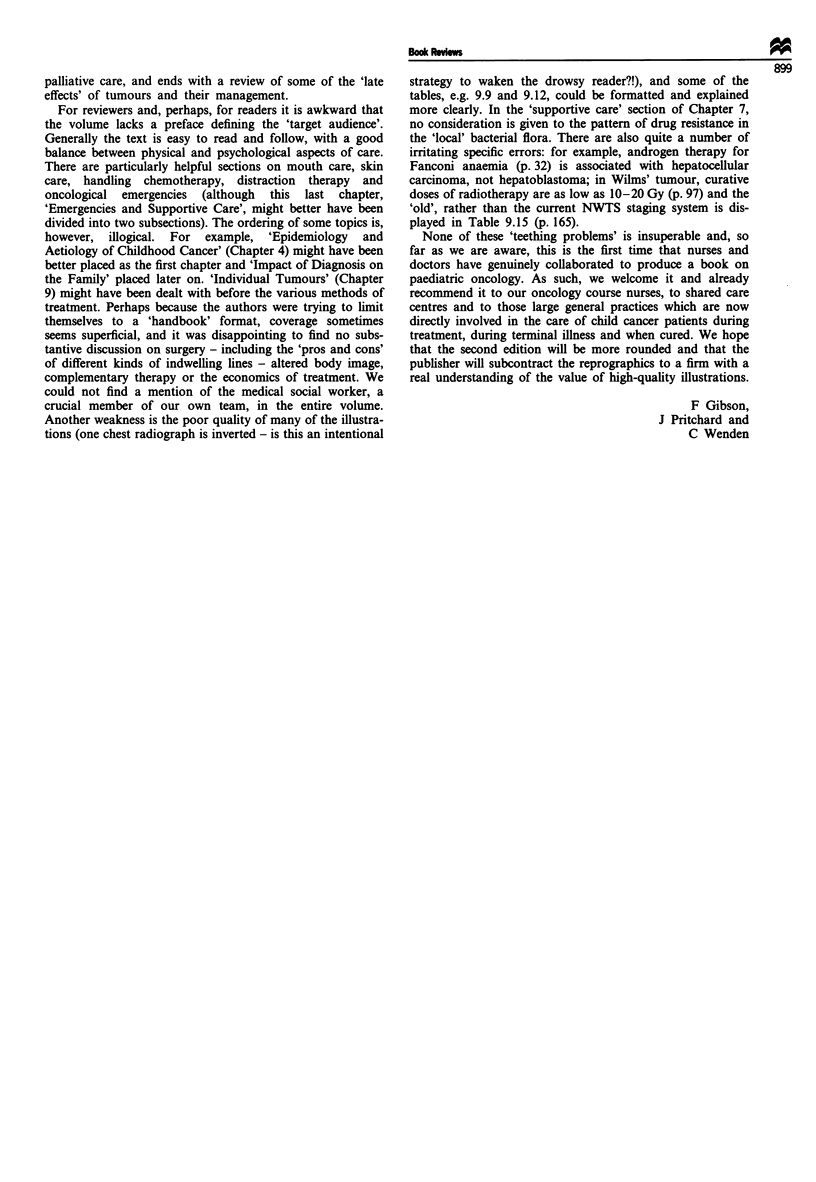# Childhood Cancer Management. A Practical Handbook

**Published:** 1995-04

**Authors:** F Gibson, J Pritchard, C Wenden


**Childhood Cancer Management. A Practical Handbook**
CR Pinkerton, P Cushing and B Sepion London: Chapman & Hall Medical, 1994, 247 pp. £22.50.

This book provides a survey of current practice in the diagnosis and management of childhood cancer, including leukaemia, in the United Kingdom. The first chapters deal with epidemiology, diagnosis and staging and the book then moves on to address various methods of treatment, including palliative care, and ends with a review of some of the 'late effects' of tumours and their management.

For reviewers and, perhaps, for readers it is awkward that the volume lacks a preface defining the 'target audience'. Generally the text is easy to read and follow, with a good balance between physical and psychological aspects of care. There are particularly helpful sections on mouth care, skin care, handling chemotherapy, distraction therapy and oncological emergencies (although this last chapter, 'Emergencies and Supportive Care', might better have been divided into two subsections). The ordering of some topics is, however, illogical. For example, 'Epidemiology and Aetiology of Childhood Cancer' (Chapter 4) might have been better placed as the first chapter and 'Impact of Diagnosis on the Family' placed later on. 'Individual Tumours' (Chapter 9) might have been dealt with before the various methods of treatment. Perhaps because the authors were trying to limit themselves to a 'handbook' format, coverage sometimes seems superficial, and it was disappointing to find no substantive discussion on surgery – including the 'pros and cons' of different kinds of indwelling lines – altered body image, complementary therapy or the economics of treatment. We could not find a mention of the medical social worker, a crucial member of our own team, in the entire volume. Another weakness is the poor quality of many of the illustrations (one chest radiograph is inverted – is this an intentional strategy to waken the drowsy reader?!), and some of the tables, e.g. 9.9 and 9.12, could be formatted and explained more clearly. In the 'supportive care' section of Chapter 7, no consideration is given to the pattern of drug resistance in the 'local' bacterial flora. There are also quite a number of irritating specific errors: for example, androgen therapy for Fanconi anaemia (p. 32) is associated with hepatocellular carcinoma, not hepatoblastoma; in Wilms' tumour, curative doses of radiotherapy are as low as 10–20 Gy (p. 97) and the 'old', rather than the current NWTS staging system is displayed in Table 9.15 (p. 165).

None of these 'teething problems' is insuperable and, so far as we are aware, this is the first time that nurses and doctors have genuinely collaborated to produce a book on paediatric oncology. As such, we welcome it and already recommend it to our oncology course nurses, to shared care centres and to those large general practices which are now directly involved in the care of child cancer patients during treatment, during terminal illness and when cured. We hope that the second edition will be more rounded and that the publisher will subcontract the reprographics to a firm with a real understanding of the value of high-quality illustrations.

F Gibson,
J Pritchard and
C Wenden